# Childhood Trauma in Spanish Women with Fibromyalgia and Depression

**DOI:** 10.1089/whr.2024.0118

**Published:** 2025-05-30

**Authors:** Anita Ribeiro, Mari Aguilera, Clara Paz, Marta Salla, Guillem Feixas

**Affiliations:** ^1^ Alumni of the Department of Cognition, Development and Psychology of Education, Faculty of Psychology, Universitat de Barcelona, Barcelona, Spain.; ^2^ Department of Cognition, Development and Psychology of Education, Faculty of Psychology, Universitat de Barcelona, Barcelona, Spain.; ^3^ The Institute of Neurosciences, Universitat de Barcelona, Barcelona, Spain.; ^4^ Neurodevelopment eHealth Lab, eHealth Center, Universitat Oberta de Catalunya, Barcelona, Spain.; ^5^ Grupo de Investigación Bienestar Salud y Sociedad, Escuela de Psicología y Educación, Universidad de Las Américas, Quito, Ecuador.; ^6^ Department of Clinical Psychology and Psychobiology, Faculty of Psychology, Universitat de Barcelona, Barcelona, Spain.

**Keywords:** cumulative trauma, chronic pain, depression Spanish sample, childhood maltreatment

## Abstract

**Introduction:**

Childhood trauma (CT) is associated with chronic widespread pain and high rates of pain sensitization, which are typical of fibromyalgia (FM), and with FM itself. The present investigation was twofold: it analyzed the prevalence of single types and cumulative types of CT in a Spanish sample of women diagnosed with FM with depressive symptoms.

**Methods:**

A reanalysis of data gathered at baseline for a randomized clinical trial of treatment methods for depression in 104 women with FM and depressive symptoms was conducted using the reanalysis data of the self-reported Childhood Trauma Questionnaire Short Form before treatment.

**Results:**

This study included higher and lower thresholds for identifying CT. Prevalence varied according to the threshold used; lower thresholds highlighted emotional neglect (52%) as higher than all other single subscales, followed by emotional abuse (42%), sexual abuse (42%), physical neglect (30%), and physical abuse (27%). At higher thresholds, emotional abuse was the highest (37%), followed by sexual abuse (31%), physical neglect (30%), physical abuse (27%), and emotional neglect (26%).

**Conclusions:**

This study’s results show that CT assessment is a necessary component of intake protocols for patients with FM.

## Introduction

Fibromyalgia (FM) is characterized by widespread chronic pain, with increased pain responses to stimuli perceived as nociceptive, with or without joint stiffness. It is accompanied by allied symptoms such as problems with sleep, memory, digestion, and headaches.^1,2^ A recent study found the prevalence of FM in adults in Spain and Europe to be significantly stable between 2000 and 2016, estimated at 2.5%, with the female sex being the variable most associated with FM, with an odds ratio of 10.156 (95% confidence interval: 5.068–20.352).^3^

Child maltreatment is the most prevalent environmental factor that increases the vulnerability to FM.^4^ In this line, Alexander et al. found in a sample of women with FM that 57% had a history of sexual or physical abuse, a group that presented significantly greater pain, fatigue, functional disability, and stress and was mainly associated with seeking health care for chronic pain.^5^ Häuser et al. showed that the comparison between patients with FM and a control group showed a significant difference in emotional and sexual abuse.^6^ Several studies have found an association between childhood trauma (CT) and FM using the Childhood Trauma Questionnaire Short Form (CTQ-SF).^4,7,8^ For instance, Filippon et al. surveyed 114 adult women with FM, and the results showed a clinically important association between CT and loss of functionality among women with FM.^9^ Interestingly, the associations were most pronounced among individuals without comorbid depression. Other studies have correlated FM with a history of trauma in adulthood and childhood.^4,10–12^

Data from official reports of maltreatment showed that children rarely suffered just one type of maltreatment or adversity, and the occurrence of multiple types of maltreatment produced a cumulative effect that led to greater vulnerability to physical harm and mental illness in adulthood.^13^ The cumulative effects of child maltreatment have also been associated with psychopathology, including depression and physical health.^14,16^ Furthermore, the combination of CT and traumatic events in adulthood substantially increases the risk of pain later in life, suggesting that a range of unresolved stressors throughout life may be more relevant to persistent pain than CT or post-traumatic stress disorder (PTSD) alone.^17^ However, there are no studies to our knowledge describing prevalence rates of cumulative CT as measured by the CTQ-SF in a large sample of women with FM with two thresholds for its severity.

While many studies focus on one or two single types of CT or cumulative adverse childhood experiences (ACE) in FM, a novel contribution of our study was to identify the specific prevalence of cumulative types of CT.

The objectives of this article are to describe the cumulative prevalence, as well as the prevalence of single types of CT, in a Spanish sample of women with FM and depressive symptoms and compare the research sample prevalence with the prevalence of CT in other female-only studies, including clinical and community samples. The hypotheses for these objectives expected the research sample to have a high prevalence rate of CT; a higher rate for single and cumulative types of CT than in community samples; and means and standard deviations (SDs) equal to or greater than a comparable clinical sample for single types of CT.

## Methodology

This research is based on baseline data collected in a two-arm, superiority, randomized controlled trial (RCT) by Aguilera et al. comparing personal construct therapy (PCT) and cognitive behavioral therapy (CBT) for the treatment of women diagnosed with FM and current depressive symptoms.^18^ We used CTQ-SF data from the RCT, collected in the baseline phase. The CTQ-SF data were not part of the primary objectives of the clinical trial and have not been used in the publications derived from that clinical trial. To use the CTQ-SF data in this secondary data investigation, an amendment was submitted to the Bioethics Committee of the Universitat de Barcelona and approved.

### Participants

Clinical samples from patients with FM and depressive symptoms were collected from public health services. The total sample size for the parent investigation was 106 women. However, for this secondary investigation, two women were excluded because they did not complete the CTQ-SF instrument during their initial assessment, resulting in a final number of 104 participants for the present study.^7^ The participants’ ages stayed within the range of 18–70 years, with a mean age of 54.58 (SD = 8.87).

Written informed consent was obtained from all participants before participation. All procedures involving human participants were performed in accordance with the ethical standards of the institutional and/or national research committee and the 1964 Helsinki Declaration and its later amendments or comparable ethical standards.

### Instruments

#### CTQ-SF and cutoffs

To collect the data at baseline, the Spanish version of the CTQ-SF was used.^19^ The CTQ-SF is a retrospective self-report inventory consisting of 28 items and has 5 distinct factors (subscales): physical, sexual, emotional abuse, and physical and emotional neglect. All items were answered on a five-point Likert scale (1 = never true, 2 = rarely true, 3 = sometimes true, 4 = often true, and 5 = very often true). The score for each subscale ranges from 5 to 25, and the CTQ-SF manual by Bernstein and Fink provides cutoff scores to classify the levels of abuse and neglect.^7^ The higher the score, the greater the severity of abuse in each subscale. The internal consistency and reliability coefficients were good to excellent for four of the five CTQ-SF subscales in the Spanish version.^19^ Table 1 shows the cutoffs defined by Bernstein and Fink and the more restrictive cutoffs defined by Walker et al. used in this investigation.^7,20,41^ For each subscale, individuals with scores in the none–minimal range were classified in the present study as negative for exposure. In the less restrictive cutoffs, all subscales were positive for exposure from low-to-severe levels; in the restrictive cutoffs, physical abuse and neglect were positive for exposure in the low-to-severe range, and emotional and sexual abuse and emotional neglect were positive for exposure in the moderate-to-severe range.^7,20^

**Table 1.** Distinct Classifications of the CTQ-SF Subscales

Subscales of maltreatment	Bernstein and Fink’s (1998)^7^ classification				Walker et al.’s (1999)^20^ cutoff values
	None to minimal	Slight to moderate	Moderate to severe	Severe to extreme	
Emotional Abuse	5–8	9–12	13–15	16–25	10–25
Physical Abuse	5–7	8–9	10–12	13–25	8–25
Sexual Abuse	5	6–7	8–12	13–25	8–25
Emotional Neglect	5–9	10–14	15–17	18–25	15–25
Physical Neglect	5–7	8–9	10–12	13–25	8–25

Adapted from Assessing childhood maltreatment at the population level in Germany: Findings and methodological challenges by H. Glaesmer, 2016; *Child and Adolescent Psychiatry and Mental Health*, 10(1):15 (https://doi.org/10.1186/s13034-016-0104-9).

CTQ-SF, Childhood Trauma Questionnaire Short Form.

Another dimension of the CT is cumulative trauma, which is a child’s exposure to more than one type (subscale) of abuse or neglect. The number of maltreatment types that met the cutoff points was considered for the analysis of the cumulative trauma scores for each individual, which ranged from 0 (cutoff points not met in any subscale) to 5 (cutoff points met in all subscales of abuse and neglect).

### Statistical analyses

The implementation of data analyses for the prevalence investigation progressed through the following steps.^7,20^ Two normative cutoffs were used to classify CT for comparison with different samples that used one or another cutoff. Walker et al.’s restrictive cutoff identified participants with moderate-to-severe abuse or neglect levels,^20^ whereas Bernstein and Fink identified those with a low-to-severe abuse or neglect history.^7^ The reverse-scored CTQ-SF items were recoded as necessary to ensure that all data were in the same format.

To calculate the rates of cumulative CT, only the more restrictive cutoff was used, and each participant scored the number of types of abuse and neglect they met the cutoff points.^20^

Descriptive statistics were used to measure the frequency of CT and compare the prevalence of single and cumulative types of CT in the research sample with the prevalence of single and cumulative types of CT in the community and clinical samples of women from other studies.^19,21^ For comparison between our sample and the clinical sample,^19^ an independent-sample *t*-test was used to analyze the performance of these two samples, with the expectation that our sample would outperform the clinical sample’s means or equal them. We used MedCalc statistical software to calculate the difference between the observed means.^22^

## Results

We compared the prevalences of our research sample with various samples from previous studies. Walker et al. assessed a community of English-speaking women aged 18–65 years who were members of a group health cooperative in the state of Washington, USA.^20^ Having access to the participants’ medical records, they were able to identify medical complaints and symptoms of women who had a history of childhood maltreatment (sexual abuse, nonsexual abuse, and neglect). They found that those women with a history of sexual maltreatment (*n* = 201) and nonsexual maltreatment (*n* = 306) had higher percentages for all clinical symptoms, but particularly higher percentages for headache, back pain, back and joint pain, and fatigue, when compared with those women without a history of childhood abuse (*n* = 698).

The sample from Iffland et al. was a reanalysis of data from a previous study, which randomly selected a sample from the general German population in 2010, aged 14–90 years, with the help of an independent service for research, methods, and analysis located in Berlin, Germany (USUMA, *Unabhängiger Service für Umfragen, Methoden und Analysen*).^23^ Unlike the original study, Iffland and colleagues used higher CTQ-SF thresholds defined by Walker et al. to examine the prevalence of CT in the sample, which included 1328 female respondents (*N* = 2500).^20,23^

Witt et al. examined a representative sample of the general German population, a total of 2510 (53.3% female) participants between 14 and 94 years old, using the CTQ-SF and collected a range of sociodemographic information.^21^

The clinical sample from Hernandez et al. included 185 inpatient and outpatient women aged between 18 and 65 years.^19^ The sample was taken from two studies in which a group of 44 participants met the criteria for dysthymia, and another subsample of 141 patients responded to the CTQ-SF as part of a validation study of this instrument and its relationship with bipolar personality disorder (BPD). This last group of 141 patients presented a more heterogeneous clinical profile: 75 patients were diagnosed with one or more personality disorders (borderline, *n* = 32; paranoid, *n* = 5; schizotypal, *n* = 2; narcissistic, *n* = 1; histrionic, *n* = 10; avoidant, *n* = 9; dependent, *n* = 2; obsessive-compulsive, *n* = 9; and unspecified, *n* = 17); 34 patients did not meet criteria for any personality disorder but had other mental illness diagnoses (major depression disorder or dysthymia, *n* = 9; anxiety disorder, *n* = 5; substance dependence disorder, *n* = 5; adjustment, *n* = 12; eating disorder, *n* = 2; and hypochondriasis, *n* = 1); the diagnoses of the remaining 32 patients (from the sample of 141 participants) were unknown.

### Prevalence of single childhood trauma

Based on Bernstein and Fink’s cutoff values, approximately 58%–73% of the sample had none/minimal levels of abuse (emotional, physical, and sexual), and 48%–70% had none or minimal levels of neglect (emotional or physical).^7^ As shown in Table 2, the highest prevalence among all subscales was emotional neglect at 52%, representing over half of the sample, followed by emotional and sexual abuse subscales at 42% prevalence. Lower prevalence percentages were recorded for physical neglect (30%) and physical abuse (27%), representing approximately a third and a fourth of our research sample, respectively. Overall, the cutoff values set by Bernstein and Fink indicated a high incidence of abuse and neglect in the research sample.^7^ In contrast, the cutoff values established by Walker et al. showed a change in viewpoint for the analysis of the impact of specific types of CT in our research sample.^20^ Emotional neglect moved from its first place in the less restrictive cutoff value to the last place within all subscales at 26% prevalence, at 11% less than emotional abuse, and the prevalence of emotional abuse came to the forefront at 37%. Sexual abuse became the second-highest prevalence with 31% of subjects reporting childhood exposure. The physical neglect percentage stayed at 30% as the third-highest prevalence in our sample, followed by physical abuse at 27%. In the lower cutoffs, emotional neglect was double the prevalence of the more restrictive cutoff set by Walker et al.^20^

**Table 2.** Prevalence Comparison Between Different Childhood Trauma Cutoff Values

CTQ-SF subscales	Research sample, *N* = 104 Cutoff values by Bernstein and Fink (1998)^7^^,^^a^	Research sample, *N* = 104 Cutoff values by Walker et al. (1999)^20^	Female nonclinical sample, *N* = 1339 Witt et al. (2017)^21^ Cutoff values by Bernstein and Fink (1998)^7^	Walker et al. (1999)^20^ *N* = 1225	Iffland et al. (2013)^23^ Female, *n* = 1328 Cutoff values by Walker et al. (1999)^20^
	Positive for exposure (%)	Positive for exposure (%)	Positive for exposure (%)	Positive for exposure (%)	Positive for exposure (%)
Emotional Abuse	42	37	20	24	11
Physical Abuse	27	27	12	14	12
Sexual Abuse	42	31	18	18	8
Emotional Neglect	52	26	39	21	13
Physical Neglect	30	30	41	12	48

^a^ Presence—low, moderate, and severe levels according to the cutoff values used.

Using the less restrictive cutoff values defined by Bernstein and Fink, our research sample presented a significantly higher prevalence of CT in those three subscales, except for the physical neglect subscale, when compared with women from the community sample analyzed by Witt et al.^7,21^ The research sample showed a 30% prevalence of physical neglect, whereas Witt et al.’s sample showed a 41% prevalence; however, this was the smallest difference (11%) between the two samples’ subscales.^21^ Taking these elements into account, compared with Witt et al.’s community sample, the prevalence is higher in the research sample for emotional abuse (22% more), physical abuse (14% more), sexual abuse (24% more), and emotional neglect (13% more), leading to marked differences of more than 20% between the two samples concerning sexual and emotional abuse subscales.^21^

Using the more restrictive cutoff values to compare our research sample with the community samples of Iffland et al. and Walker et al.,^20,23^ our research sample had significantly higher percentages on four and five subscales of CT, respectively (Table 2). Compared with the sample of Iffland et al.,^23^ the differences were higher except for physical neglect, in which our sample prevalence was 18% less. Our sample had a 26% higher prevalence for emotional abuse, 15% for physical abuse, 23% for sexual abuse, and 13% for emotional neglect. Compared with the sample of Walker et al.,^20^ the research sample prevalence was 13% higher for emotional, physical, and sexual abuse; 15% higher for emotional neglect; and 18% for physical neglect, the largest difference between the two samples.

When comparing the clinical sample of Hernandez et al. with the research sample using means and SDs for both samples (see Table 3), the clinical sample of Hernandez et al. obtained significantly higher scores on the emotional, physical, and sexual abuse subscales.^19^ A comparison of emotional and physical neglect scores showed no significant differences between the two samples.

**Table 3.** Descriptive Statistics of Single Childhood Trauma in the Research Sample and Hernandez et al.’s (2012)^19^ and Independent *t*-Test Results

CTQ-SF subscales	Research sample *N* = 104		Spanish clinical sample (Hernandez et al., 2012)^19^ *N* = 185		Statistic
	Mean	SD	Mean	SD	*t*
Emotional Abuse	9.45	4.94	13.1	6.09	5.22***
Physical Abuse	7.23	3.7	8.44	4.89	2.19*
Sexual Abuse	7.48	4.15	9.08	5.76	2.49*
Emotional Neglect	11.34	5.31	12.47	5.46	1.71
Physical Neglect	7.32	3.44	7.5	3.28	0.44

*p ≤ 0.05; ***p < 0.001.

SD, standard deviation.

### Prevalence of cumulative childhood trauma

The cumulative CT prevalence in the research sample according to the threshold criteria defined by Bernstein and Fink and Walker et al. was compared (Table 4) with the sample from Walker et al.^7,20^

**Table 4.** Comparison Between the Research Sample’s Cumulative Childhood Trauma and Walker et al.’s Sample (1999)^20^ Using Two Cutoffs

Number of CT types	Research sample Cutoff by Bernstein and Fink (1998)^7^^,a^ *N* = 104 (%)	Research sample Cutoff by Walker et al. (1999)^20^ *N* = 104 (%)	Walker et al. (1999)^20^ *N* = 1225 (%)
0	22	39	57
1	29	25	20
2	16	10	10
3	9	10	7
4	14	9	4
5	10	9	2

^a^ Presence—low, moderate, and severe levels according to the cutoffs used.

CT, childhood trauma.

Using the least restrictive criteria,^7^ the highest percentage referred to participants who suffered a single type of CT (29%), followed by those who did not suffer any type of CT (22%). Next, the highest percentage referred to 16% of participants who experienced two types of CT, followed by 14% of participants who experienced four types of CT. Those who experienced three and five types of CT were 9% and 10%, respectively, and three types of CT had the lowest percentage.

Changing the threshold criteria to a restrictive cutoff value (Table 2),^20^ our sample showed a higher percentage of participants without any CT experience (39%), followed by 25% of participants who experienced a single type of CT. The percentages of participants who experienced two and three types of CT were 10%, and four and five types were 9%. The percentage of Walker et al.’s sample that had no CT exposure was 57%, which was 18% higher than this research sample.^20^ Although the cumulative of two types of CT was equal in the community sample of Walker et al., three, four, and five cumulative types of CT were higher in our sample.

## Discussion

Our research sample of women with FM with depressive symptoms had a considerably higher prevalence of CT in at least four subscales of CT for both lower and higher cutoff criteria than the community samples, confirming our hypothesis. Some of our findings confirmed the findings of a previous study that found a high prevalence of CT in FM. Filippon et al. studied the prevalence of CT in a sample of 114 women with FM, using the low severity level cutoffs defined by Bernstein and Fink, and found a considerably higher prevalence of CT than our sample in all subscales: emotional neglect (75.4%), physical neglect (75.4%), emotional abuse (69.3%), physical abuse (54.4%), and sexual abuse (35.1%).^7,9^ One hundred ten individuals (96.5%) reported at least one type of CT.^9^

While Witt et al. analyzed their study data based on restrictive cutoff values,^20,21^ the study provided numbers and percentages for each CT subscale across both sex threshold criteria, which allowed for the calculation of percentages at the least restrictive cutoff values^7^ for women (Table 2). Using these less restrictive cutoff values, our research sample showed a significantly higher prevalence of CT across all subscales except the physical neglect subscale compared with the Witt et al. sample.^21^

The community sample of Witt et al. had a higher prevalence of physical neglect than our research sample when compared with both severity cutoffs; however, the physical neglect subscale has been shown to have internal consistency problems, which calls for caution when assessing that subscale.^21^ Our sample had a higher prevalence of emotional abuse (22% higher), physical abuse (15% higher), sexual abuse (plus 24%), and emotional neglect (13% higher), leading to marked differences in more than 20% between the two samples concerning the sexual and emotional abuse subscales.

The two studies by Iffland et al. and Walker et al. used for comparison with our sample at more restrictive cutoffs were separated by more than a decade and cultural differences, which may explain some of the differences in prevalence between these two community samples, as awareness of CT developed with time.^20,23^

At less restrictive cutoff values,^7^ there is a risk of accepting levels of impact of abuse or neglect that may not be representative of traumatic experiences. At more restrictive cutoff values,^20^ there is a risk of missing nuances of abuse and neglect, which can be traumatic for more sensitive or vulnerable individuals. However, higher thresholds more accurately discriminate levels of maltreatment that may cause trauma or psychological harm, owing to their intensity, frequency, and duration, ensuring that the CTQ-SF assessment powerfully establishes CT.

Emotional neglect and abuse were the most intense CT subscales assessed in this study (Table 3), as defined by their mean and SD. These scales assess the impact on a child’s sense of worth or well-being when ignored, dismissed, or subjected to humiliation, degradation, intimidation, ridicule, or threats.^24,25^ Emotional abuse can be directed toward a child’s affections, such as physical aggression toward the child’s pet or outright punitive separation from the pet, or toward a child’s friends, such as a lack of respect and belittling when speaking of them. Emotional neglect does not recognize the child’s emotions, and the child is not validated in childhood. In these cases, it becomes difficult for adults with a history of CT to identify and understand their own emotions and those of others.

Emotional abuse and neglect have been studied as possible influences on “self-organization disorders,” a set of behaviors found in complex post-traumatic stress disorder (CPTSD).^26^ Self-organization disorders negatively affect the behavioral capacity to regulate emotions, leading to dysregulation, which can generate somatization of these effects, a negative self-concept, and rupture or conflict in relationships.

Spertus et al. found that emotional abuse and neglect predict anxiety, depression, physical symptoms, and exposure to other traumas throughout life.^25^ They concluded that emotional abuse and neglect are subtle forms of trauma and potent predictors of emotional functioning in adulthood. Additionally, this study found that emotional abuse and neglect were significantly correlated with the number of doctor visits and the direct impact of childhood emotional experiences on adult physical health.

On the sexual abuse subscale of the CTQ-SF, our survey sample showed approximately double the prevalence rate (low threshold criteria applied) compared with community samples. Even when restrictive threshold values were applied to the research sample, the percentage of sexual abuse maintained a high percentage difference from community samples. These results confirmed literature findings implicating sexual abuse with chronic pelvic pain and chronic widespread pain, such as in FM, or both, and other various types of pain, creating a “pain-prone phenotype.”^27–29^ Perhaps the type, frequency, chronicity, or extent of sexual abuse affects the type of pain, inviting investigations into the nature of sexual abuse concerning painful symptomatology.^30^

Physical abuse was highly prevalent in our research sample and may have occurred in conjunction with other forms of abuse or neglect, perhaps increasing the cumulative effect of CT. A history of physical abuse has been associated with the development of antisocial personality traits and paranoid or narcissistic personality traits, and is also related to behavioral externalization.^31–33^ A history of physical abuse favors conduct disorder or oppositional defiant disorder and appears to be related to pain.^5,16,34^ Gota et al. studied a mixed sample of 593 consecutive FM patients, 87% female; and 223 of these patients reported childhood abuse, 155 (69.5%) physical abuse, 125 (56.5%) sexual abuse, and 78 (34.9%) both types of abuse.^36^

Comparing the rates between our research sample and the clinical sample of Hernandez et al.,^19^ the values were close but not equal or higher, as expected. Emotional and physical neglect were remarkably similar between the two groups. The comparison between studies emphasized the emotional neglect subscale, which had the highest mean in our research sample and the second-highest mean in Hernandez et al.^19^ The findings, with marginal differences in the abuse subscales, particularly the emotional abuse subscale, showed higher mean scores in Hernandez et al.^19^ These differences between the two studies may have occurred because the clinical sample included in Hernandez et al. presented with severe mental illnesses, and none presented with FM.^19^

Our results indicate a trend that would likely be reinforced by a larger sample, particularly the trend toward larger cumulative CT effects (three or more types). The accumulation of more than one type of childhood maltreatment, that is, a combination of physical, sexual, or emotional maltreatment types (abuse and neglect), is associated with an increased risk of FM,^34,37^ chronic pelvic pain,^30^ and other types of pain, including emotional pain.^15^ Some research focused on cumulative ACE, considering the dose-dependent relationship between the number of adverse events (including CT) and chronic pain.^38^

This study had some limitations. One of them refers to the inclusion criteria for depressive symptoms, which may have filtered those who had exposure to CT but did not present with depressive symptoms at the time of screening. Regarding the CTQ-SF, it relies on retrospective self-reports, the validity of which is subject to recall and response biases, such as appraisals of what constitutes maltreatment that might still be based on a distorted view of “right and wrong” inherited from the childhood environment. In addition, other studies have found remarkable variations in the percentage of documented survivors of childhood sexual abuse who remember the abuse as adults.^39^ Therefore, recollecting past abuse data is subject to omissions and errors, implying that measurements taken using retrospective instruments are likely to underreport CT.

The CTQ-SF data used for this investigation did not include the three questions of this instrument that screen for the minimization of maltreatment due to statistical logistics and limitations. Minimizing this information could have improved the ability to discern participants who failed to report CT findings. In addition, regarding the CTQ-SF, there was an extremely low Cronbach’s alpha index of internal consistency for two subscales, sexual abuse (*α* = 0.41) and physical neglect (*α* = 0.19), which need to be addressed in future studies, perhaps with a test–retest before and after treatment.

In addition to the above limitations, because CT is implicated in a wide range of psychiatric diagnoses,^40^ some items in the exclusion criteria of this investigation might have excluded a large number of women with FM who had a history of manic or hypomanic episodes, current substance abuse, and those who were receiving psychological therapy at the time of screening. Finally, many studies did not have available information about the effect of cumulative CT on their samples to compare with this research sample, or if they had, their samples did not have similar characteristics to our research sample; for example, they displayed data of samples of mixed sex, which made it challenging to establish more comparisons.

## Conclusion

In conclusion, the comparison between the prevalence of CT in our sample and community samples showed that our sample presented a higher number and greater levels of severity for four out of five types of abuse and neglect incidents, and a higher rate of cumulative CT compared with community samples. This result encourages the inclusion of CT evaluation as a necessary component of assessment protocols for patients with FM, warranting treatment approaches that address the history of CT in this population.

## Abbreviations Used

ACE: adverse childhood experiences
BPD: bipolar personality disorder
CBT: Cognitive Behavioral Therapy
CI: confidence interval
CPTSD: complex post-traumatic stress disorder
CT: childhood trauma
CTQ-SF: childhood trauma questionnaire-short form
FM: fibromyalgia
n: partial population
N: total population
PCT: Personal Construct Therapy
PTSD: post-traumatic stress disorder
RCT: randomized controlled trial
SD: standard deviation
USUMA: Unabhängiger Service für Umfragen, Methoden und Analysen
